# Three Weeks of rTMS Treatment Maintains Clinical Improvement But Not Electrophysiological Changes in Patients With Depression: A 6-Week Follow-Up Pilot Study

**DOI:** 10.3389/fpsyt.2019.00351

**Published:** 2019-06-07

**Authors:** Kyung Mook Choi, Soo-Hee Choi, Sang Min Lee, Kuk-In Jang, Jeong-Ho Chae

**Affiliations:** ^1^Institute for Brain and Cognitive Engineering, Korea University, Seoul, South Korea; ^2^Department of Psychiatry, Seoul St. Mary’s Hospital, Seoul, South Korea; ^3^Institute of Biomedical Industry, Catholic University of Korea, College of Medicine, Seoul, South Korea; ^4^Department of Biomedicine & Health Sciences, Catholic University of Korea, College of Medicine, Seoul, South Korea; ^5^Department of Psychiatry, Seoul National University Hospital, Seoul, South Korea

**Keywords:** rTMS, depression, maintenance effects, event-related potential, standardized low-resolution brain electromagnetic tomography, emotion regulation

## Abstract

Our previous study demonstrated that 3 weeks of repetitive transcranial magnetic stimulation (rTMS) increases P200 amplitudes and improves the symptoms of depression and anxiety in depression patients. In the present study, we investigated whether 3 weeks of rTMS treatment maintained the P200 amplitude in patients with depression at 6 weeks of follow-up. We measured the 6-week maintenance effects of rTMS using clinical questionnaires and an auditory oddball paradigm. Twenty-one patients with medication-resistant major depression participated in this pilot study. All patients underwent rTMS treatment for 3 weeks; they completed clinical ratings and performed the auditory oddball task at the pre-treatment, post-treatment, and 6-week follow-up visit (3 weeks after finishing rTMS treatment). The results revealed an increase in P200 amplitudes as well as improvements in the symptoms of depression and anxiety by 3 weeks of rTMS treatment. Furthermore, the results demonstrated maintenance effects on clinical ratings at 6-week follow-up. Depression and anxiety scales showed improvements in post-treatment and maintenance effects at the 6-week follow-up. Although P200 amplitude showed a significant main effect for 3 time points (baseline, post-treatment, and 6-week follow-up visit), at 2 time point comparisons, P200 amplitudes significantly increased in post-treatment compared to those of the baseline condition but did not show the maintenance effects of long-term rTMS at the 6-week follow-up compared to those of the baseline condition (  *p* = .173, Bonferroni correction). Standardized low-resolution brain electromagnetic tomography (sLORETA) for P200 showed significant activation in the left middle frontal gyrus in post-treatment but no significant activation at the 6-week follow-up. Moreover, the amplitudes of overall topographic distribution were reduced at 6 weeks of follow-up. The 3 weeks of rTMS treatment induced the maintenance of the improvements in the symptoms of depression and anxiety. However, considering the results of the event-related potential (ERP) and sLORETA, 3 weeks of rTMS treatment may not be sufficient to maintain this improvement, implying that a treatment period of more than 3 weeks may be required to reveal the electrophysiological maintenance effect of rTMS.

## Introduction

Repetitive transcranial magnetic stimulation (rTMS) has been used for treatment of mood disorders, and the therapeutic effects of that have been demonstrated in many studies. In particular, rTMS has been recognized as an effective method in the treatment of medication-resistant major depression (1–3). Our previous study (3) demonstrated that 3 weeks of rTMS increases P200 amplitudes as well as improves depression and anxiety symptoms in patients with depression. In the present study, we tried to analyze whether 3 weeks of rTMS treatment maintains the increased P200 amplitude as well as whether it improves depression and anxiety symptoms in patients with depression. We measured 6 weeks of maintenance effects of rTMS using clinical questionnaires and auditory oddball event-related potential (ERP) after 3 weeks of rTMS treatment. In our previous study (3), 3 weeks of rTMS over the left dorsolateral prefrontal cortex (DLPFC) increased P200 amplitudes as well as improved clinical ratings, compared to baseline observations without any rTMS treatment. P200 is known to be related to task relevance evaluation of stimulus items, such as enhancing relevant features or suppressing irrelevant features (4). Based on the properties of P200, we interpreted that the increase in P200 amplitude after 3 weeks of rTMS treatment may be related to enhancing positive stimuli or suppressing negative stimuli during improvement of symptoms in patients with depression, which could thereby result in a positive attitude during everyday life events (3).

HF-rTMS over the left DLPFC showed the modulation of functional connectivity in the frontostriatal network–related therapeutic effects in depression patients (5). HF-rTMS increased the functional connectivity of the DLPFC, caudate, and globus pallidus in the left hemisphere, and the limbic circuit of both hemispheres (6). Accelerated HF-rTMS also caused a negative correlation between the left superior medial prefrontal cortex (PFC) and the anterior cingulate cortex (ACC) (7). The results of Baeken et al. suggested that the stronger negative correlation between the ACC and left mPFC might be indicative of a beneficial outcome of accelerated HF-rTMS, in that it can predict clinical effects. Therefore, HF-rTMS can modulate the functional connectivity of the brain. In a previous study, prolonged exposure to rTMS induced changes in hypoperfusion results using positron emission tomography (PET) (8); this showed that long-term rTMS (10 daily sessions) over the left PFC induced changes in depression-related symptoms that were inversely correlated between 20-Hz and 1-Hz rTMS; 20-Hz rTMS showed a superior ability to improve baseline hypoperfusion. Additionally, in a study that examined changes in regional cerebral blood flow (rCBF) using PET during rTMS, 20-Hz rTMS applied for 2 weeks over the left PFC showed an increase in rCBF in the PFC, cingulate gyrus, and amygdala in the left hemisphere, and in the hippocampus, parahippocampus, insula, thalamus, basal ganglia, uncus, and cerebellum bilaterally, whereas 1-Hz rTMS over the left PFC showed a decrease in rCBF in small areas of the right PFC, left basal ganglia, and left amygdala, and left medial temporal cortex (9). A recent study (10) reported that a direct single-pulse TMS to the left DLPFC using concurrent TMS–fMRI in healthy participants triggered activity of the subgenual (sg) ACC associated with the improvement of depressive symptoms in patients with major depression (11, 12), showing that TMS to the DLPFC can propagate to the sgACC. A study by Vink et al. (10) showed that rTMS treatment effects in patients with major depression can be studied more extensively using a concurrent TMS–fMRI method.

There has been no study reporting the maintenance effects of rTMS after long-term rTMS treatment without additional rTMS treatment using ERP, although previous studies reported the clinical improvement effects of rTMS using depression scales in depression patients (3, 13, 14). In the present study, we expected that the rTMS effects shown in 3 weeks of rTMS treatment would be maintained after 6 weeks’ time, even without additional rTMS treatment. That is, we intended to observe whether the P200 amplitudes increased by 3 weeks of rTMS treatment can be maintained for 6 weeks’ time without further rTMS treatment after the first 3 weeks. We hypothesized that 3 weeks of treatment would show maintenance effects in the 6-week follow-up. For the depression patients investigated in our previous study (3) and newly added patients, we measured 6-week maintenance effects of rTMS using clinical rating scales and auditory oddball ERP in this 6-week follow-up pilot study.

## Methods

### Participants

Thirty patients diagnosed with major depression (15) were enrolled at the Department of Psychiatry, Catholic University of Korea. They suffered from medication-resistant major depression (refractory to at least two different classes of antidepressants). Rating scales and ERP were measured for them at baseline (pre-treatment). However, nine of the patients were excluded due to pain from the rTMS stimuli (six patients), a personal situation (one patient), hospitalization (one patient), and absence from the 6-week follow-up appointment (one patient). Therefore, 21 patients (8 males) participated at baseline, at 3 weeks (post-treatment), and at 6-week follow-up. Among the 21 patients in this study, 17 had participated in our previous study (3); 1 of the 18 patients from the previous study was excluded because of the lack of a 6-week follow-up. Moreover, four patients were newly recruited in this study. All patients who completed the trial were outpatients during the trial. Their mean age was 36.5 years old (standard deviation, 14.7 years; range, 19–67 years). All patients received pharmacotherapy for depression before the start of the rTMS treatment, but drugs or dosages were not changed during the 3-week rTMS treatment and till the 6-week follow-up. All the patients who used medications or antidepressants continued to maintain dosages at stable levels without any changes throughout the rTMS treatment. Medications in use included the following: amitriptyline, escitalopram, fluoxetine, mirtazapine, paroxetine, and sertraline (3). Patients with neurological illness, substance abuse, major head trauma, seizure, or pacemakers or hearing aids were excluded.

We received from all participants signed informed consent forms that were approved by the Institutional Review Board of the Catholic University of Korea prior to their participation in the study.

### rTMS Procedure

The rTMS procedure used in the present study was the same as that in our previous study (3). We used a TAMAS stimulator with a figure-8 coil (REMED, Daejon, Korea) that was applied to the left DLPFC of all the patients (13) with the following parameters: intensity, 110% of the resting motor threshold of the right abductor pollicis brevis muscle; frequency, 10 Hz for 5 s; intertrain interval, 25 s. Each treatment session lasted for 30 min, including 60 trains and 3,000 pulses, and was repeated for 5 days every week till a period of 3 weeks (a total of 45,000 pulses for 15 treatment sessions).

### Rating Scales

Patients were tested before and 3 weeks after the start of the rTMS treatment and at the follow-up visit (6 weeks) using the same rating scales as in our previous study (3), namely, the Hamilton Depression Rating Scale (HAM-D, 17 items) (16), Beck Depression Inventory (BDI) (17), Hamilton Anxiety Scale (HAM-A) (18), State–Trait Anxiety Inventory (SAI for state anxiety and TAI for trait anxiety) (19), Emotion Regulation Questionnaire (ERQ) (20), Cognitive Emotion Regulation Questionnaire (CERQ) (21), and Ruminative Response Scale (RRS) (22).

### Electroencephalography Recording and Analyses

Electrophysiological recording was performed before and 3 weeks after the start of the rTMS treatment, as well as at the follow-up visit (6 weeks), using the same methods and instruments as in our previous study (3). We presented the auditory oddball task using E-Prime (Psychology Software Tools, Pittsburgh, PA, USA) and recorded and amplified EEG activity using a NeuroScan NuAmps amplifier (Compumedics USA, Ltd., El Paso, TX, USA) from 34 positions (FP1, FP2, Fz, F3, F4, F7, F8, FCz, FC3, FC4, FT7, FT8, FT9, FT10, Cz, C3, C4, T3, T4, CPz, CP3, CP4, TP7, TP8, Pz, P3, P4, T5, T6, Oz, O1, O2, PO1, and PO2). Subsequently, we performed ERP analyses using NeuroScan 4.5 software (Compumedics USA, Ltd., Charlotte, NC, USA). Details of electroencephalography (EEG) recording and analyses in the present study were the same as in our previous study (3). We measured peak amplitudes and latencies of N100, P200, N200, and P300 from the peaks between 100 and 180 ms, 180 and 260 ms, 240 and 350 ms, and 320 and 500 ms, respectively, for the target tones at Fz, FCz, Cz, Pz, FP1, and FP2. The mean numbers of accepted epochs among the 60 target tones’ epochs were 54.8 ± 7.6, 51.7 ± 8.3, and 51.8 ± 6.9, at baseline, 3 weeks, and 6 weeks, respectively.

### Statistical Analyses

**Rating scales and ERP oddball tasks** **:** We performed all statistical analyses using Predictive Analytics Software (PASW) version 18.0 (SPSS, Inc., Chicago, USA), as in our previous study (3). The rating scales were analyzed using the repeated-measures analysis of variance (ANOVA) and paired *t*-test to compare relevant time points (baseline, 3 weeks, and 6 weeks). And then we used Bonferroni correction for a *post hoc* test of repeated-measures ANOVA. The ERP oddball tasks were analyzed using repeated-measures ANOVA with six electrode sites and time of measurement (baseline, 3 weeks, and 6 weeks) as the within-subject factors. As rTMS was applied to the PFC, as described in our previous study (3), we analyzed six electrode sites (Fz, FCz, Cz, Pz, FP1, and FP2). We evaluated the sphericity assumption using Mauchley’s test and used the Greenhouse–Geisser correction to evaluate the *F* ratios in order to control Type 1 error in the repeated-measures design.

**Source analysis** **:** The source analysis using a statistical nonparametric mapping method was performed for source activation of the ERP waveform and standardized low-resolution brain electromagnetic tomography (*sLORETA*) (23–25). We conducted a *t*-statistic on the log-transformed data with subject-wise normalization. Statistical significance was assessed nonparametrically with a randomization test (n = 5,000) that corrects for multiple comparisons. Thirty-four channels were used for the sLORETA: FP1, FP2, Fz, F3, F4, F7, F8, FCz, FC3, FC4, FT7, FT8, FT9, FT10, Cz, C3, C4, T3, T4, CPz, CP3, CP4, TP7, TP8, Pz, P3, P4, T5, T6, Oz, O1, O2, PO1, and PO2.

## Results

The rating scales were analyzed for the 21 patients. The ERP tasks were analyzed for 14 patients after seven patients were excluded due to an excess number of artifacts at 3 or 6 weeks. We excluded the cases with an excess number of artifacts at either 3 or 6 weeks, and therefore, the rejected cases are relatively high in ERP analyses.

### Rating Scales

The clinical rating scales are shown in **Table 1**. For the HAM-D, the main effect of time of measurement was significant [*F*(2,40) = 10.12, *p* = .0003]. The HAM-D decreased significantly in post-treatment [*t*(20) = 3.82, *p* = .003, Bonferroni correction], and there was no significant difference between post-treatment and the 6-week follow-up [*t*(20) = −1.01, *p* = .971, Bonferroni correction]. The HAM-D decreased significantly in the 6-week follow-up after rTMS treatment, compared to pre-treatment [*t*(20) = 3.14, *p* = .015, Bonferroni correction]. For the HAM-A, the main effect of time of measurement was significant [*F*(2,40) = 10.52, *p* = .0002]. The HAM-A decreased significantly in post-treatment [*t*(20) = 3.49, *p* = .007, Bonferroni correction], and there was no significant difference between post-treatment and the 6-week follow-up [*t*(20) = 0.16, *p* = .1.000, Bonferroni correction]. The HAM-A decreased significantly in the 6-week follow-up after rTMS treatment, compared to pre-treatment [*t*(20) = 4.40, *p* = .001, Bonferroni correction]. For the BDI, the main effect of time of measurement was significant [*F*(2,40) = 15.62, *p* = .00001]. The BDI decreased significantly in post-treatment [*t*(20) = 4.20, *p* = .001, Bonferroni correction], and there was no significant difference between post-treatment and the 6-week follow-up [*t*(20) = 0.37, *p* = 1.000, Bonferroni correction]. The BDI decreased significantly in the 6-week follow-up after rTMS treatment, compared to pre-treatment [*t*(20) = 5.04, *p* = .0002, Bonferroni correction]. For the SAI, the main effect of time of measurement was significant [*F*(2,40) = 5.45, *p* = .008]. The SAI decreased significantly in post-treatment [*t*(20) = 3.77, *p* = .004, Bonferroni correction], and there was no significant difference between post-treatment and 6-week follow-up [*t*(20) = 0.02, *p* = 1.000, Bonferroni correction]. The SAI did not decrease at 6-week follow-up after rTMS treatment compared to pre-treatment [*t*(20) = 2.52, *p* = .061, Bonferroni correction]. In contrast, for the TAI, the main effect of time of measurement was not significant [*F*(2,40) = 1.28, *p* = .289].

**Table 1:** Clinical ratings for 3 time points (baseline, 3 weeks, 6 weeks) and statistical results.

		Pre-treatment	Post-treatment (3 weeks)	6-week follow-up	Repeated-measures ANOVA				Pairwise comparisons (post hoc test)^†^													
									Pre–post					Post–6-week follow-up				Pre–6-week follow-up				
	*N*	Mean ± SD	Mean ± SD	Mean ± SD	*F*	*df*	*p*		*t*	*df*	*p*		*d*^#^	*t*	*df*	*p*	*d*^#^	*t*	*df*	*p*		*d*^#^
**HAM-D**	21	16.5 ± 6.4	11.5 ± 4.5	12.4 ± 4.7	10.12	2,40	.0003	**	3.82	20	,003	**	.904	–1.01	20	.971	.196	3.14	20	.015	*	.730
**HAM-A**	21	17.2 ± 7.2	12.4 ± 5.9	12.2 ± 6.0	10.52	2,40	.0002	**	3.49	20	.007	**	.729	0.16	20	1.000	.034	4.40	20	.001	**	.754
**BDI**	21	24.4 ± 11.4	17.0 ± 10.2	16.4 ± 8.1	15.62	2,40	.00001	**	4.20	20	.001	**	.684	0.37	20	1.000	.065	5.04	20	.0002	**	.809
**SAI**	21	59.2 ± 8.1	53.8 ± 10.1	53.8 ± 9.8	5.45	2,40	.008	**	3.77	20	.004	**	.590	0.02	20	1.000	.000	2.52	20	.061		.601
**TAI**	21	60.1 ± 10.0	58.0 ± 10.5	59.5 ± 11.2	1.28	2,40	.289		1.72	20	.302		.205	–1.09	20	.870	.138	0.43	20	1.000		.057
**RRS**	21	64.5 ± 12.4	61.7 ± 13.7	62.4 ± 12.9	2.06	2,40	.141		1.74	20	.294		.214	–0.54	20	1.000	.053	1.51	20	.436		.166
**ERQ—reappraisal**	21	2.9 ± 1.2	3.2 ± 1.2	3.2 ± 1.4	1.26	2,40	.294		–1.55	20	.408		.250	0.09	20	1.000	.000	–1.54	20	.420		.230
**ERQ—suppression**	21	4.0 ± 1.0	4.0 ± 1.2	3.8 ± 1.0	1.02	2,40	.368		–0.45	20	1.000		.000	1.30	20	.623	.181	1.03	20	.945		.200
**Self-blame**	21	12.4 ± 5.0	11.6 ± 4.7	11.0 ± 3.7	1.46	2,40	.245		0.99	20	1.000		.165	0.73	20	1.000	.142	1.74	20	.292		.318
**Acceptance**	21	12.4 ± 3.4	12.1 ± 3.3	11.7 ± 3.8	0.36	2,40	.634		0.28	20	1.000		.090	0.79	20	1.000	.112	0.78	20	1.000		.194
**Focus on thought/rumination**	21	13.5 ± 4.2	12.3 ± 4.6	11.8 ± 4.0	3.64	2,40	.035	*	1.74	20	.290		.272	0.77	20	1.000	.116	2.86	20	.029	*	.415
**Positive refocusing**	21	7.5 ± 2.8	9.0 ± 3.4	8.6 ± 3.9	3.58	2,40	.037	*	–2.49	20	.065		.482	0.80	20	1.000	.109	–1.79	20	.267		.324
**Refocus on planning**	21	11.2 ± 3.8	12.6 ± 3.4	11.8 ± 4.4	2.62	2,40	.085		–2.43	20	.074		.388	1.30	20	.625	.203	–0.90	20	1.000		.146
**Positive reappraisal**	21	10.0 ± 2.9	10.3 ± 3.6	9.5 ± 4.7	0.65	2,40	.525		–0.62	20	1.000		.092	1.05	20	.921	.191	0.60	20	1.000		.128
**Putting into perspective**	21	10.7 ± 3.9	10.7 ± 3.8	11.0 ± 4.3	0.18	2,40	.834		–0.09	20	1.000		.000	–0.43	20	1.000	.074	–0.60	20	1.000		.073
**Catastrophizing**	21	12.3 ± 4.0	11.5 ± 4.1	11.5 ± 4.1	1.04	2,40	.364		1.17	20	.766		.198	0.07	20	1.000	.000	1.49	20	.452		.198
**Blaming others**	21	11.3 ± 4.5	10.2 ± 4.4	10.9 ± 4.9	1.64	2,40	.206		1.85	20	.239		.247	–1.15	20	.789	.150	0.66	20	1.000		.085

*p < 0.05, **p < 0.01, ^†^Bonferroni correction, ^#^Cohen’s d effect size.

The RRS and ERQ (reappraisal and suppression) did not show any significant effect for time of measurement (statistical results shown in **Table 1**). In CERQ, for the strategies of acceptance, refocus on planning, positive reappraisal, putting into perspective, catastrophizing, self-blame, and blaming others, the main effects of time of measurement were not significant. However, for the strategy of focus on thought/rumination, the main effect of time of measurement was significant [*F*(2,40) = 3.64, *p* = .035]. Although the strategy of focus on thought/rumination did not decrease significantly at post-treatment compared to pre-treatment, and there was no significant difference between post-treatment and the 6-week follow-up (*p* = .290 and *p* = 1.000, respectively, Bonferroni correction), there was a significant decrease observed during the 6-week follow-up after rTMS treatment when compared to pre-treatment (*p* = .029, Bonferroni correction). For the positive refocusing strategy, the main effect of time of measurement was significant [*F*(2,40) = 3.58, *p* = .037]. The positive refocusing strategy did not decrease significantly in post-treatment (*p* = .065, Bonferroni correction), and there was no significant difference between post-treatment and 6-week follow-up (*p* = 1.000, Bonferroni correction). The positive refocusing strategy also did not show a significant decrease at the 6-week follow-up after rTMS treatment as compared to pre-treatment (*p* = .267, Bonferroni correction).

### ERP Auditory Oddball Task

**Figure 1:**
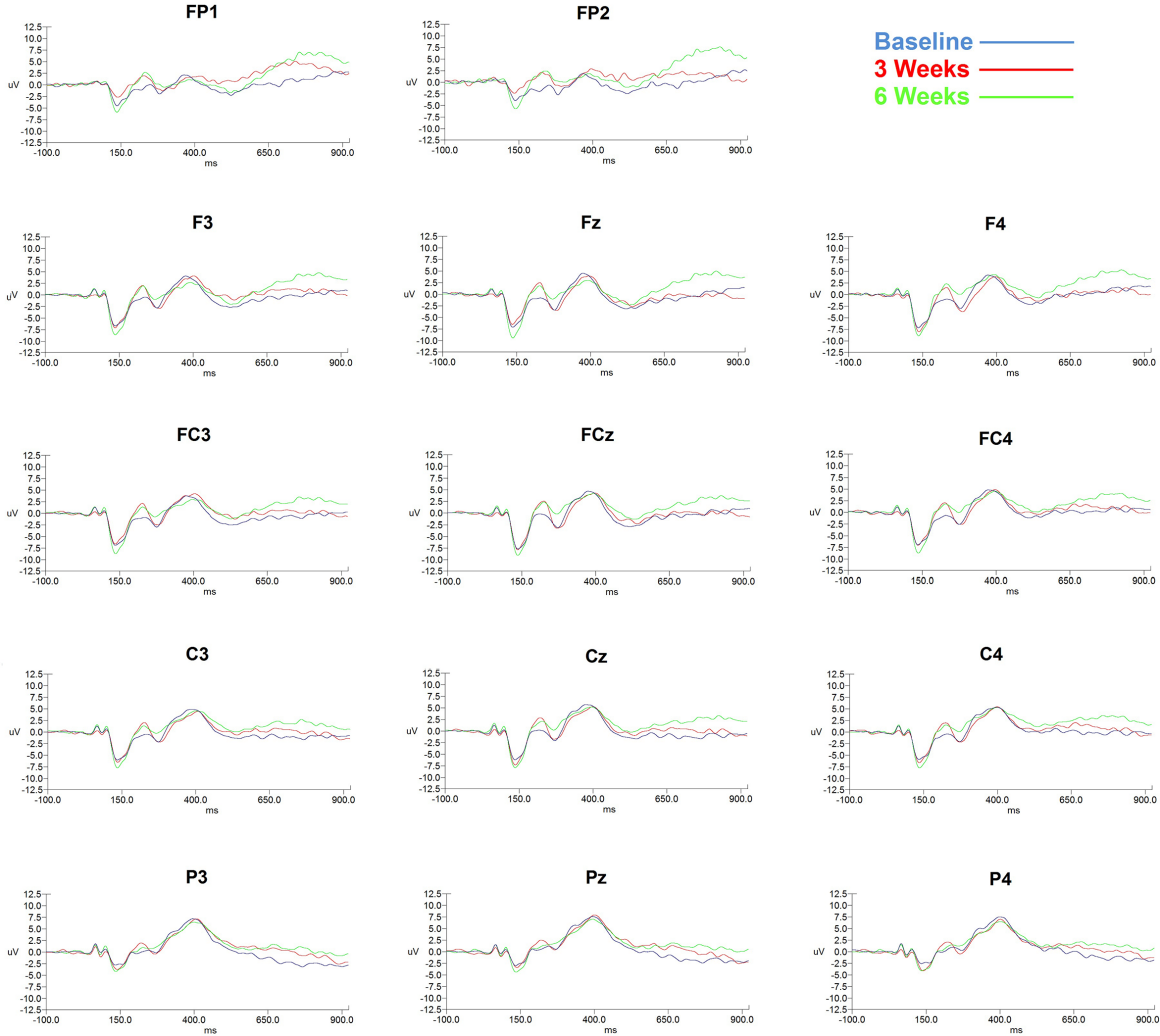
ERP changes shown in 14 channels according to 3 time points (baseline, 3 weeks, and 6 weeks).

The Fz, FCz, Cz, Pz, FP1, and FP2 channels and time of measurement were submitted to repeated-measures ANOVA (**Figures 1** **–** **3** and **Table 2**).

**Figure 2:**
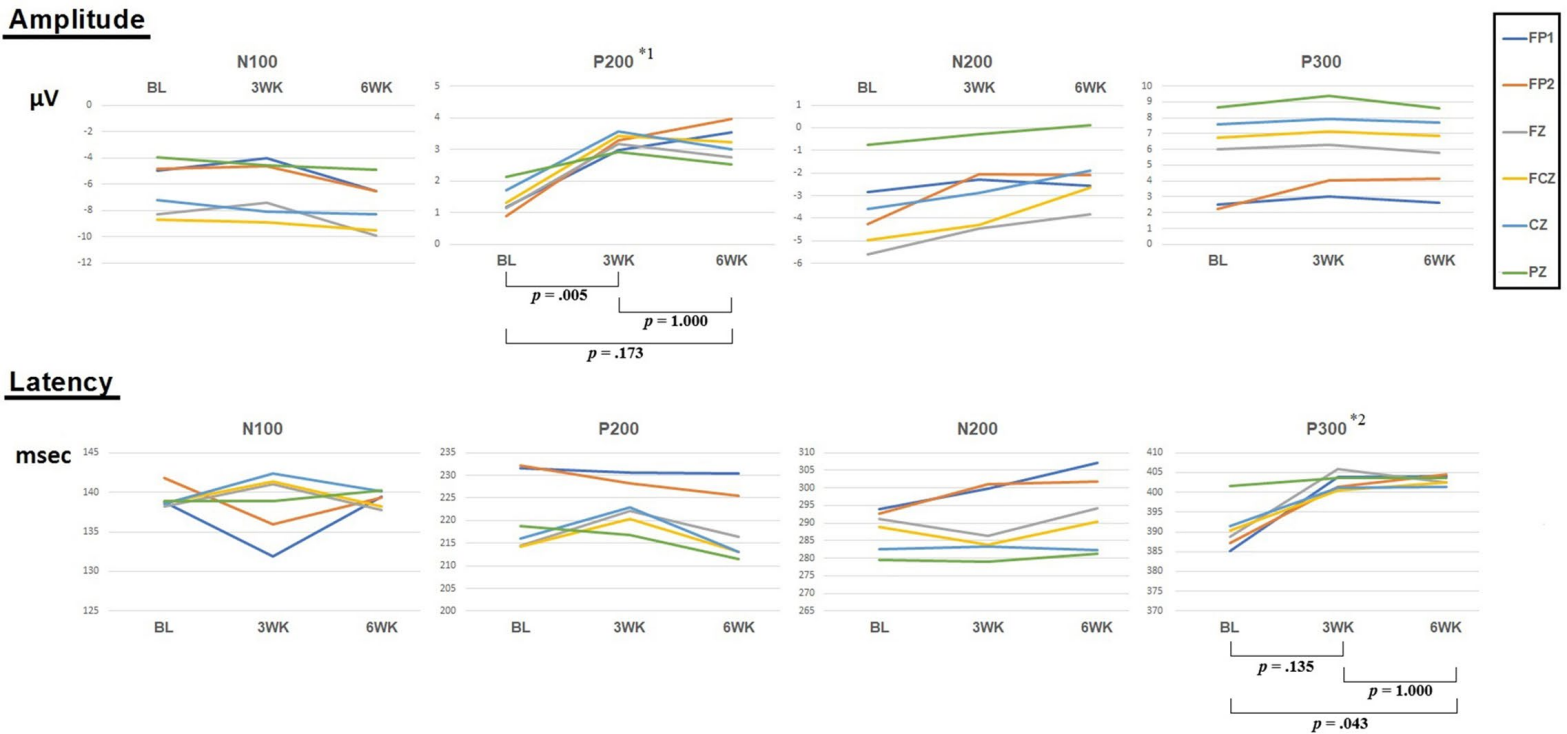
Amplitudes and latencies of four components (N100, P200, N200, and P300) according to 3 time points (baseline, 3 weeks, 6 weeks). *^1^: *F* = 3.82, *p* = .035. *^2^: *F* = 3.87, *p* = .034. *P*-values indicate Bonferroni correction for *post hoc* test of repeated-measures ANOVA.

**Figure 3:**
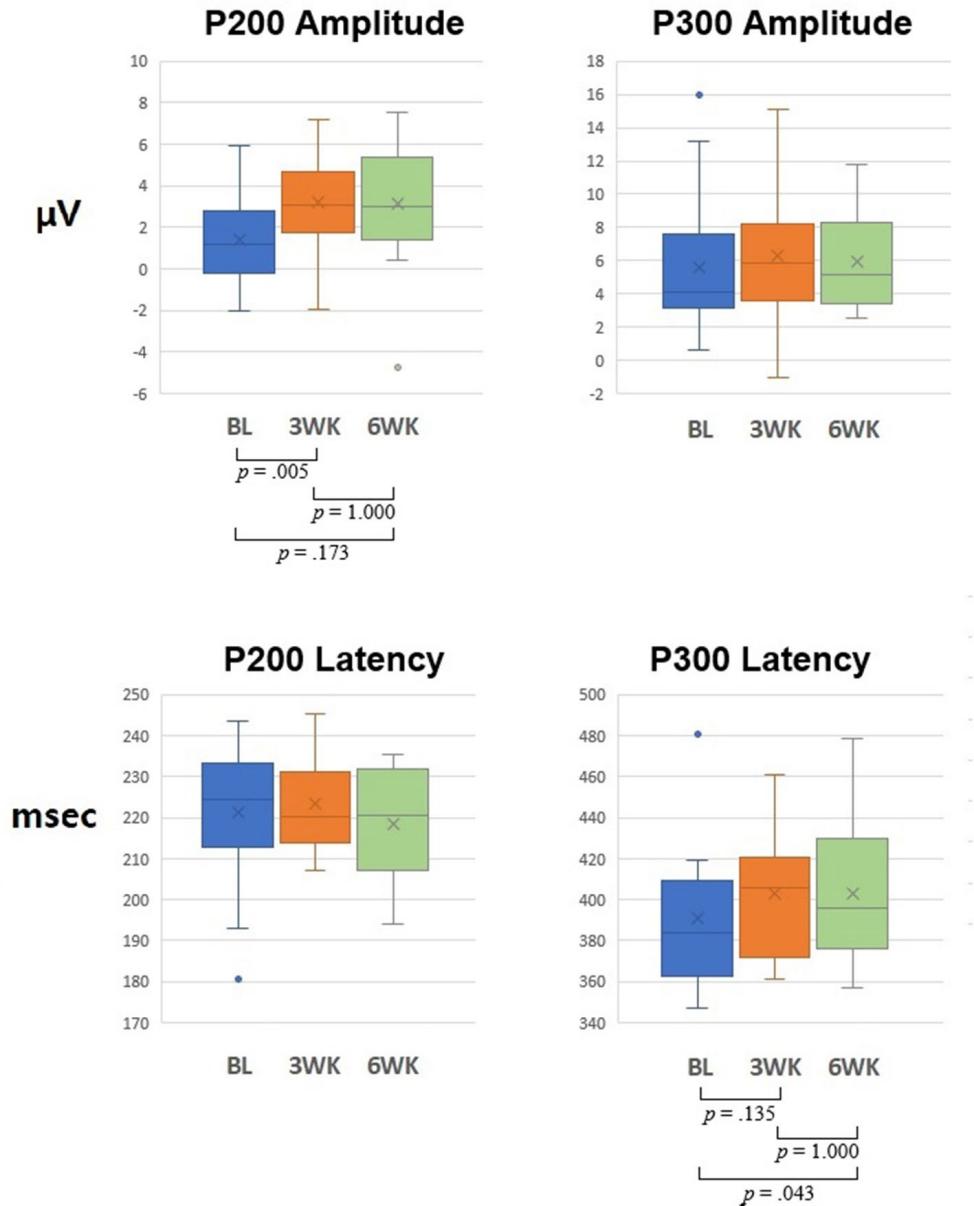
Graph of 6 channels’ (FP1, FP2, Fz, FCz, Cz, and Pz) mean for P200 and P300 amplitudes and latencies at baseline, 3-week, and 6-week time points. *P*-values indicate Bonferroni correction for *post hoc* test of repeated-measures ANOVA.

**Table 2:** Means and standard deviations of the amplitudes and latencies of the N100, P200, N200, and P300 components at the FP1, FP2, Fz, FCz, Cz, and Pz channels.

Amplitude												
	N100 (Mean ± SD)			P200 (Mean ± SD) *^1^			N200 (Mean ± SD)			P300 (Mean ± SD)		
	BL	3WK	6WK	BL	3WK	6WK	BL	3WK	6WK	BL	3WK	6WK
**FP1**	–5.0 ± 4.0	–4.0 ± 2.5	–6.6 ± 2.8	1.2 ± 3.1	3.0 ± 3.0	3.5 ± 2.6	–2.8 ± 3.1	–2.3 ± 3.1	–2.6 ± 3.0	2.7 ± 5.0	3.0 ± 3.2	2.6 ± 3.7
**FP2**	–4.9 ± 4.6	–4.6 ± 1.8	–6.6 ± 3.2	0.9 ± 3.4	3.3 ± 3.1	4.0 ± 2.9	–4.2 ± 4.4	–2.0 ± 4.0	–2.1 ± 4.0	2.6 ± 5.9	4.0 ± 4.9	4.2 ± 4.4
**FZ**	–8.3 ± 4.7	–7.4 ± 2.9	–9.9 ± 3.5	1.1 ± 3.1	3.2 ± 3.4	2.7 ± 4.7	–5.6 ± 4.3	–4.5 ± 7.3	–3.8 ± 4.9	6.0 ± 4.9	6.3 ± 5.5	5.8 ± 3.7
**FCZ**	–8.7 ± 5.1	–8.9 ± 3.4	–9.5 ± 3.7	1.3 ± 3.5	3.4 ± 3.5	3.2 ± 4.8	–4.9 ± 5.3	–4.3 ± 7.2	–2.6 ± 5.5	6.7 ± 4.9	7.1 ± 5.5	6.8 ± 3.3
**CZ**	–7.2 ± 4.2	–8.1 ± 3.5	–8.3 ± 3.8	1.7 ± 3.5	3.6 ± 3.5	3.0 ± 4.6	–3.6 ± 5.7	–2.9 ± 6.3	–1.9 ± 5.4	7.6 ± 5.2	7.9 ± 5.3	7.7 ± 3.0
**PZ**	–4.0 ± 2.7	–4.6 ± 2.6	–4.9 ± 2.2	2.1 ± 3.0	2.9 ± 2.5	2.5 ± 3.1	–0.7 ± 4.0	–0.3 ± 4.8	0.1 ± 2.9	8.6 ± 6.0	9.4 ± 5.2	8.6 ± 3.2
Latency												
	N100 (Mean ± SD)			P200 (Mean ± SD)			N200 (Mean ± SD)			P300 (Mean ± SD) *^2^		
	BL	3WK	6WK	BL	3WK	6WK	BL	3WK	6WK	BL	3WK	6WK
**FP1**	139.5 ± 13.7	131.9 ± 14.3	139.5 ± 10.6	231.5 ± 24.7	230.6 ± 22.0	230.4 ± 16.3	294.4 ± 31.5	299.7 ± 19.8	307.0 ± 29.9	386.5 ± 36.0	403.9 ± 28.2	404.0 ± 30.4
**FP2**	142.4 ± 16.5	136.0 ± 15.1	139.4 ± 10.8	232.2 ± 24.3	228.1 ± 20.9	225.6 ± 18.8	293.1 ± 31.0	301.0 ± 17.6	301.9 ± 30.9	388.6 ± 33.0	401.4 ± 24.2	404.6 ± 35.5
**FZ**	138.2 ± 15.1	141.0 ± 12.2	137.8 ± 7.3	214.5 ± 18.7	222.1 ± 12.8	216.4 ± 16.1	291.1 ± 29.9	286.4 ± 18.9	294.3 ± 33.1	388.8 ± 38.7	405.8 ± 34.6	402.4 ± 38.9
**FCZ**	138.6 ± 13.9	141.4 ± 11.8	138.2 ± 6.8	214.3 ± 18.2	220.4 ± 13.3	213.0 ± 15.4	288.8 ± 31.4	283.9 ± 20.9	290.4 ± 34.7	390.4 ± 42.9	400.6 ± 36.5	402.4 ± 38.9
**CZ**	138.6 ± 13.9	142.4 ± 11.4	140.1 ± 9.5	216.0 ± 18.9	223.0 ± 14.1	213.1 ± 16.6	282.6 ± 26.1	283.3 ± 21.8	282.2 ± 32.0	391.4 ± 42.9	401.1 ± 37.1	401.4 ± 38.3
**PZ**	138.9 ± 16.0	138.9 ± 15.7	140.3 ± 12.4	218.7 ± 22.7	216.8 ± 18.4	211.5 ± 21.1	279.4 ± 28.1	279.1 ± 23.8	281.4 ± 32.8	401.6 ± 33.4	403.6 ± 23.4	403.7 ± 36.8

*1: F = 3.82, p = .035. *2: F = 3.87, p = .034.

**Amplitude**: In ANOVA of 3 time points (baseline, 3 weeks, and 6 weeks), for the N100 amplitude, the main effect of time of measurement was not significant [*F*(2,26) = 2.169, *p* = .135], but demonstrated a significant main effect for the electrode site [*F*(5,65) = 16.392, *p* = .0005]. There was no significant interaction between the time of measurement and the electrode site [*F*(10,130) = 1.683, *p* = .181]. For the P200 amplitude, the main effect of time of measurement was significant [*F*(2,26) = 4.209, *p* = .026], but no significant main effect for the electrode site was observed [*F*(5,65) = .066, *p* = .898]. There was no significant interaction between time of measurement and electrode site [*F*(10,130) = 1.267, *p* = .297]. For pairwise comparisons of 2 time points, P200 amplitude showed a significant difference for the time of measurement between baseline and 3 weeks [*t*(13) = −4.004, *p* = .005, Bonferroni correction, Cohen’s *d* = .835] and did not show a significant difference for the time of measurement between 3 and 6 weeks [*t*(13) = .063, *p* = 1.000, Bonferroni correction, Cohen’s *d* = .000]. That is, P200 amplitude at 3 weeks was higher than that at the baseline and did not show a difference from that at 6 weeks. However, P200 amplitude did not show a significant difference for the time of measurement between baseline and 6 weeks [*t*(13) = −2.081, *p* = .173, Bonferroni correction, Cohen’s *d* = .675]. For the N200 amplitude, the main effect of time of measurement was not significant [*F*(2,26) = 2.160, *p* = .153], but a significant main effect for the electrode site was detected [*F*(5,65) = 6.849, *p* = .007]. There was no significant interaction between the time of measurement and the electrode site [*F*(10,130) = 1.316, *p* = .280]. For the P300 amplitude, the main effect of time of measurement was not significant [*F*(2,26) = .171, *p* = .843], but a significant main effect for the electrode site was observed [*F*(5,65) = 18.856, *p* = .00002]. There was no significant interaction between the time of measurement and the electrode site [*F*(10,130) = .398, *p* = .756].

**Latency**: ANOVA of 3 time points (baseline, 3 weeks, and 6 weeks) for the N100 amplitude showed that the main effect of time of measurement was not significant [*F*(2,26) = .027, *p* = 923], and that of the electrode site was not significant [*F*(5,65) = .503, *p* = .663]. There was no significant interaction between the time of measurement and the electrode site [*F*(10,130) = 1.497, *p* = .237]. For the P200 amplitude, the main effect of time of measurement was not significant [*F*(2,26) = 1.026, *p* = .373], but a significant main effect for the electrode site was observed [*F*(5,65) = 6.617, *p* = .009]. There was no significant interaction between the time of measurement and the electrode site [*F*(10,130) = 1.285, *p* = .285]. For the N200 amplitude, the main effect of time of measurement was not significant [*F*(2,26) = .878, *p* = .427], but a significant main effect for the electrode site was observed [*F*(5,65) = 6.111, *p* = .010]. There was no significant interaction between the time of measurement and the electrode site [*F*(10,130) = 1.454, *p* = .233]. For the P300 amplitude, the main effect of time of measurement was significant [*F*(2,26) = 4.330, *p* = .024], but there was no significant main effect for the electrode site [*F*(5.65) = .639, *p* = .542]. There was no significant interaction between the time of measurement and the electrode site [*F*(10,130) = 1.704, *p* = .147]. For pairwise comparisons of 2 time points, P300 latency showed a significant difference for the time of measurement between baseline and 3 weeks, [*t*(13) = −2.219, *p* = .135, Bonferroni correction, Cohen’s *d* = .371] and did not show a significant difference for the time of measurement between 3 weeks and 6 weeks [*t*(13) = −.082, *p* = 1.000, Bonferroni correction, Cohen’s *d* = .012]. P300 latency showed a significant difference for the time of measurement between baseline and 6 weeks [*t*(13) = −2.826, *p* = .043, Bonferroni correction, Cohen’s *d* = .355].

### Topographic Maps

**Figure 4:**
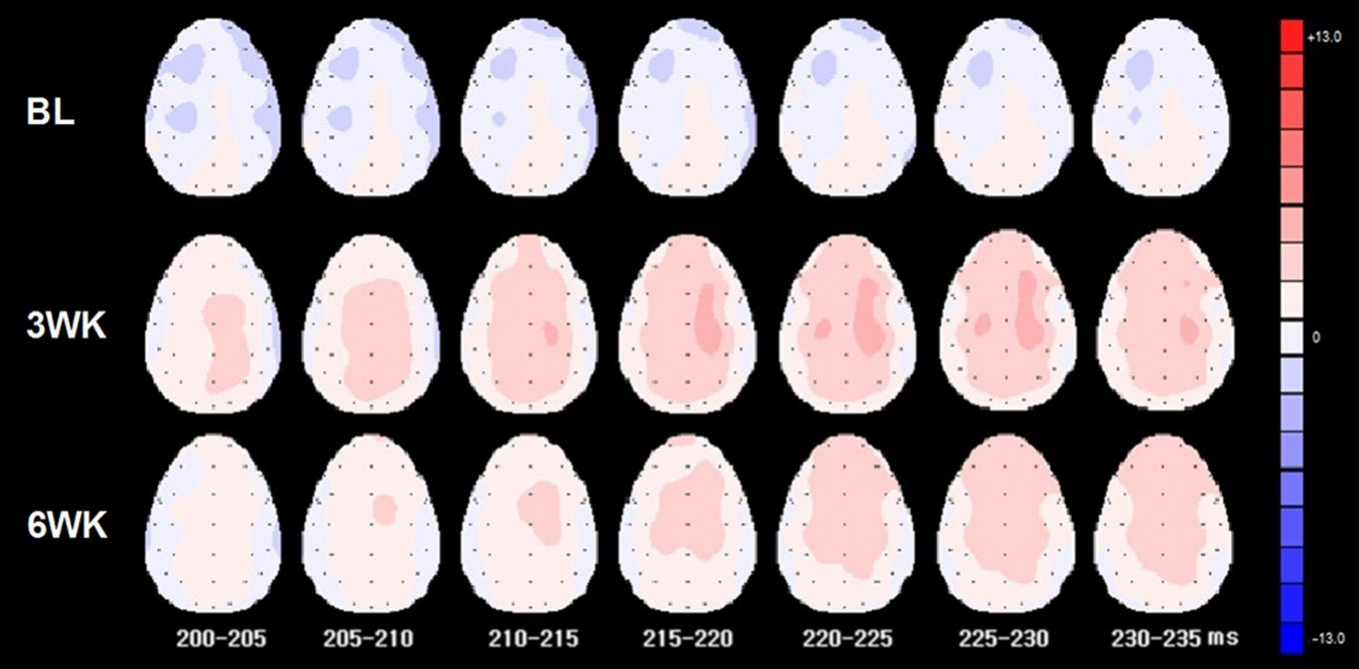
Topographic maps of 3 time points (baseline, 3 weeks, 6 weeks) for P200 from 200 to 235 ms.

The changes in P200 amplitude at 3 time points (baseline, 3 weeks, and 6 weeks) are presented in **Figure 4**. As shown in the topographic changes, overall, P200 amplitude increased saliently in post-treatment but decreased at 6 weeks compared to 3 weeks.

### sLORETA

**Figure 5:**
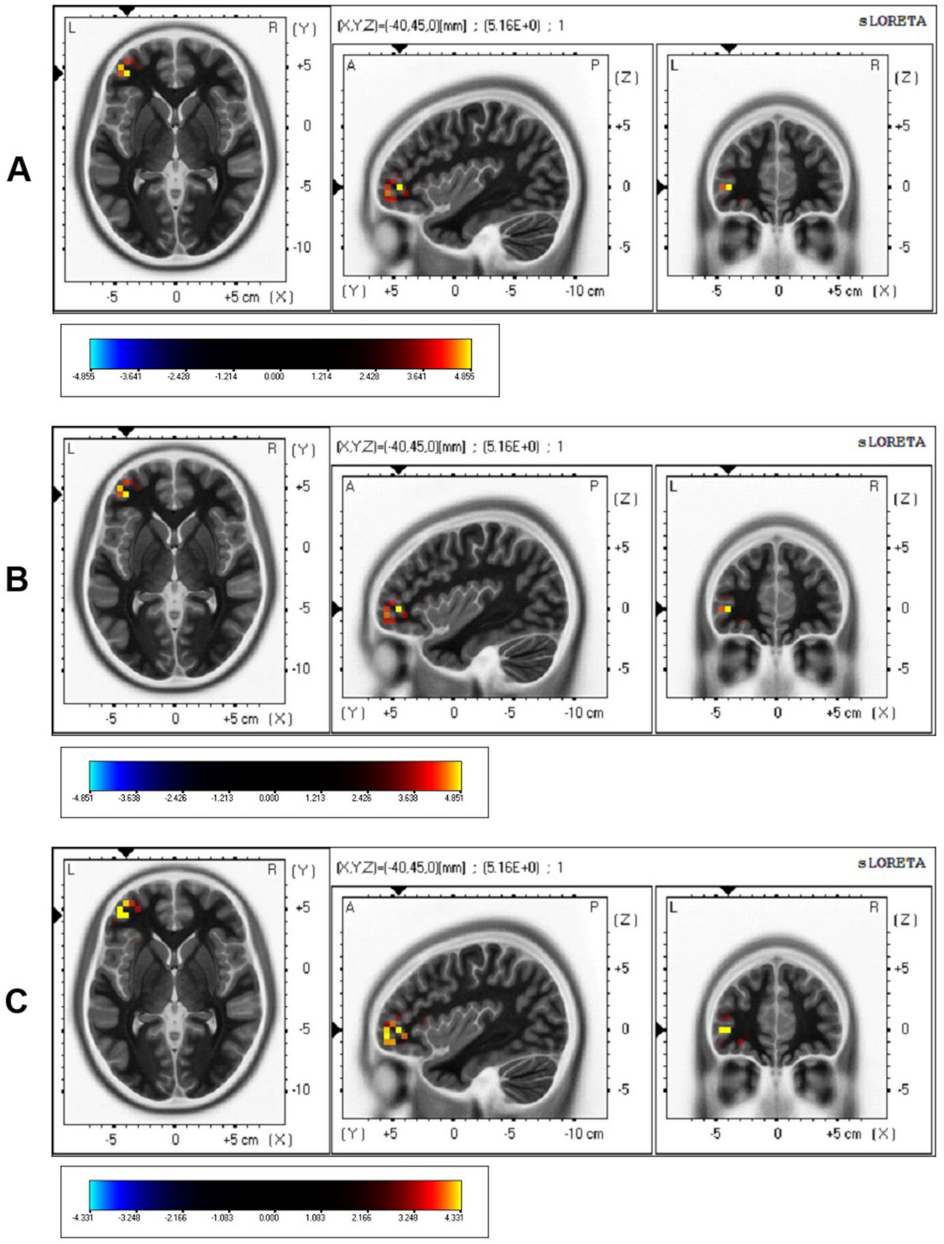
sLORETA of 3 weeks minus baseline. **(A)** *t* = 4.855, *p* < 1.0, two-tailed; **(B)** *t* = 4.851, *p* < .05, one-tailed; **(C)** *t* = 4.331, *p* < 1.0, one-tailed.

Source localization was analyzed for the P200 component that was observed to be a significant effect of time in the ANOVA. The P200 latency was analyzed for source localization (**Figure 5**). The sLORETA for the P200 latency showed a significant activation in the middle frontal gyrus (MFG) at 3 weeks, compared to that at the baseline (*t* = 4.855, *p* < 1.0, two-tailed; *t* = 4.851, *p* < .05, one-tailed; *t* = 4.331, one-tailed, *p* < 1.0), although there was no significant region at *p* < .05, two-tailed (**Table 3** and **Figure 4**). However, the sLORETA for the P200 latency at 6 weeks showed no significant activation compared to that at 3 weeks (all *ps* > .05, for two-tailed and one-tailed), and the sLORETA for the P200 latency at 6 weeks showed no significant activation compared to the baseline (all *p*’s > .05, for two-tailed and one-tailed). Montreal Neurological Institute (MNI) coordinates of source localization showed activation in the left MFG (location stimulated by rTMS) at 3 weeks compared to the baseline (*t* = 5.164, *p* < .1, two-tailed; *p* < .05, one-tailed) (**Table 3**).

**Table 3:** Montreal Neurological Institute (MNI) coordinates of source localization at 3 weeks compared to those at baseline.

Region	BA*	MNI coordinate			*t*-value	*p* 2-tailed	*p* 1-tailed
		X	Y	Z			
Middle frontal gyrus (left)	10	–40	45	0	5.164	*p* < .1	*p* < .05
Inferior frontal gyrus (left)	10	–45	50	0	4.721		*p* < .1
Middle frontal gyrus (left)	10	–40	55	–5	4.476		*p* < .1
Inferior frontal gyrus (left)	10	–45	45	0	4.378		*p* < .1
Middle frontal gyrus (left)	11	–30	40	–5	4.338		*p* < .1

*BA, Brodmann area.

## Discussion

In the previous study (3), we reported that 3 weeks of rTMS treatment induced significant electrophysiological changes (i.e., P200 component of ERP oddball task) accompanied by the improvement of symptoms of depression and anxiety in patients with depression. In the present pilot study, we replicated and extended the previous study’s results (3). By extending the previous study to investigate the maintenance effects, we measured the maintenance effects at the 6-week follow-up and in an increased number of patients. The results showed an increase in P200 amplitudes as well as the improvement of the symptoms of depression and anxiety. The results demonstrated maintenance effects on clinical ratings, although the 3 weeks of rTMS treatment ceased before the 3 weeks when follow-up effects were measured. We hypothesized that 3 weeks of treatment could show maintenance effects at 6-week follow-up. However, P200 amplitudes significantly increased compared to those of the baseline condition but did not show the maintenance effects of long-term rTMS at the 6-week follow-up. That is, although there was no significant difference of the amplitudes between 3 weeks (post-treatment) and 6 weeks (follow-up), the difference of the amplitudes between baseline (pre-treatment) and 6 weeks (follow-up) was not significant (*p* = .173, Bonferroni correction), and the amplitudes of the overall topographic distribution were reduced at the 6-week follow-up compared to those at 3 weeks. In addition, sLORETA for P200 showed significant activation in the left MFG during the 3 weeks of rTMS treatment when compared with 3 weeks and the baseline, but no significant activation at the 6-week follow-up when compared with 6 weeks and baseline.

P200 amplitude showed a significant main effect for the time of measurement for ANOVA of 3 time points (baseline, 3 weeks, and 6 weeks). P200 amplitude at 3 weeks was significantly higher than that at baseline and did not show a difference with that at 6 weeks. However, P200 amplitude did not show a significant difference between baseline and 6 weeks, which indicates that the P200 amplitude at 6 weeks decreased more than that at 3 weeks. P300 latency showed a significant main effect for time of measurement for ANOVA of 3 time points (baseline, 3 weeks, and 6 weeks). P300 latency showed a significant difference between baseline and 6 weeks. Two previous studies investigating ERP changes in patients with depression demonstrated that long-term rTMS increased P200 amplitude (3, 26). Of the studies, that by Spronk et al. (26) had some limitations, which included heterogeneous sessions, a small number of subjects, and the resultant marginal significance, compared to the results of Choi et al. (3). The study of Spronk et al. (26) also showed that other components of post-treatment ERP differ from those of baseline, which may have been due to the limitations of that study. In the present study, we found that the ERP results analyzed in ANOVA of 14 subjects confirmed our previous study (3).

In the clinical rating scales, depression and anxiety were decreased after 3 weeks of rTMS treatment, and these improvements were maintained at the 6-week follow-up. Therefore, depression and anxiety rated by HAM-D, HAM-A, and BDI showed improvement in post-treatment and maintenance effects at the 6-week follow-up. The results of improvement in depression and anxiety indicate the treatment and maintenance effects of 3 weeks of rTMS. Previous studies, including ours, have shown the improvement effects of long-term rTMS (3, 13, 14, 26), and in particular, the present study demonstrated the maintenance effects of 3 weeks of rTMS. As for state and trait anxiety scales, the SAI decreased in post-treatment, and there was no significant difference between post-treatment and 6-week follow-up. The SAI did not decrease in the 6-week follow-up after rTMS treatment, compared to pre-treatment. That is, the improvement of SAI was not maintained after 3 weeks of rTMS treatment. On the other hand, the TAI did not show an effect for time of measurement. These results replicated our previous results (3) by showing that state anxiety is improved by 3 weeks of rTMS but trait anxiety is not improved by 3 weeks of rTMS. Interestingly, the improvement of SAI by 3 weeks of rTMS was not maintained at the 6-week follow-up, indicating that the improvement of state anxiety did not show a long-term effect. The results of SAI and TAI in the present study replicated our previous study’s results (3) and extended to the verification of maintenance effects, which suggests that 3 weeks of rTMS treatment influences state anxiety but not trait anxiety from the perspective of post-treatment, but does not influence state anxiety and trait anxiety from the perspective of long-term effects. This finding, using SAI and TAI in the present study, combined with the conclusions of our previous study, is interesting in that the improvement of anxiety by 3 weeks of rTMS is differentiated between state and trait anxiety, which has not been reported before as far as we know. Future studies may need to investigate the neural mechanism for this difference in the improvement of anxiety by long-term rTMS treatment in patients with depression.

With respect to emotion regulation scales using CERQ, for the strategies of refocus on planning, acceptance, putting into perspective, positive reappraisal, catastrophizing, self-blame, and blaming others, the main effects of time of measurement were not significant. However, for the strategies of focus on thought/rumination and positive refocusing, the main effects of time of measurement were significant. Regarding two factors of CERQ, although in the case of positive refocusing, there were no significant differences between post-treatment and pre-treatment, between post-treatment and 6-week follow-up, and between 6-week follow-up and pre-treatment on the *post hoc* test (*p* = .065, *p* = 1.000, and *p* = .267, respectively), in the case of the strategy of focus on thought/rumination, there were no significant decreases between post-treatment and pre-treatment and between post-treatment and 6-week follow-up, but there was a significant decrease between the 6-week follow-up and pre-treatment on the *post hoc* test (*p* = .290, *p* = 1.000, and *p* = .029, respectively). In particular, the strategies of focus on thought/rumination and positive refocusing showed an inverse trend after rTMS treatment. That is, on average, the scores of focus on thought/rumination (negative emotion regulation) decreased, and those of the positive refocusing strategy (positive emotion regulation) increased at post-treatment and 6-week follow-up compared to baseline (**Table 1**). Overall, the results of emotion regulation ratings using CERQ showed that there were partial treatment effects of emotion regulation factors in patients with depression by 3 weeks of rTMS treatment. In concordance with the results indicating that there were no changes in trait anxiety as per the post-treatment and maintenance effects by 3 weeks of rTMS treatment, emotion regulation factors except the strategies of focus on thought/rumination and positive refocusing may be related to trait facets of emotion regulation. Because there has been no study so far, except our previous study (3), using emotion regulation scales in rTMS treatment of medication-resistant major depression, our results using long-term rTMS treatment could have very important implications relevant to rTMS treatment in patients with depression, considering the relationship between CERQ and depressive symptoms (27).

In the topographic map of amplitude shown in the present study, overall amplitudes at 6 weeks decreased compared to those at 3 weeks, which reflects the reduction of physiological effects of 3 weeks of rTMS. The reason for the reduction of overall amplitudes could be that the maintenance effects were not sustained by 3 weeks of rTMS treatment in the depression patients, which reflects that a 3-week period of rTMS treatment is not enough to maintain the treatment effect. Therefore, a 3-week period of rTMS may not be enough to maintain electrophysiology shown in ERP. Future studies may need to demonstrate longer-term treatment studies (i.e., 4 to 8 weeks) or may need to include a longitudinal study with longer and more frequent intervals to identify the maintenance effect of long-term rTMS treatment. Regarding the sLORETA, the results for the P200 latency showed significant activation in the MFG at 3 weeks, compared to that at baseline (**Figure 4**). The sLORETA for the P200 latency at 6 weeks showed no significant activation compared to that at 3 weeks and showed no significant activation compared to the baseline. There may be some reasons for there being no significant difference in 6 weeks in sLORETA. Although the 3 weeks of rTMS treatment maintained clinical scale scores that were almost the same at 6 weeks as at 3 weeks, the 3-week rTMS may not be enough to produce sufficient brain plasticity, as shown by the ERP and sLORETA results. On the other hand, the improvement of the clinical scale scores may also gradually appear even after a decrease in electrophysiology. Usually, typical rTMS treatment for medication-resistant depression has been recommended to be applied for 4 to 8 weeks (14). Therefore, longer rTMS treatment beyond 3 weeks and measurement may be needed in order to identify the electrophysiological maintenance effect of rTMS.

The present study has some limitations. First, this study compared treatment effects and maintenance effects according to time without a sham group. Therefore, a further study including a sham group will be needed in the future. Second, we used sLORETA to analyze the source level, which has limitations in the exact change and maintenance effects of source by rTMS treatment.

In conclusion, 3 weeks of rTMS induces improved depression and anxiety symptoms. However, as shown in our results of electrophysiology at 6 weeks, although improvement of clinical ratings was maintained by 3 weeks of rTMS treatment, 3 weeks of rTMS may not be long enough to maintain the improvement in the brain region’s activation. These results of our 6-week follow-up pilot study may indicate that the electrophysiological decrease is followed by a decrease in clinical ratings. Our results suggest that longer rTMS treatment beyond 3 weeks and measurement may be needed to identify the electrophysiological maintenance effect of rTMS.

## Data Availability Statement

The datasets generated for this study are available on request to the corresponding author.

## Ethics Statement

All subjects signed a written informed consent form that was approved by the Institutional Review Board of Catholic University of Korea prior to their participation in the study.

## Author Contributions

KC and J-HC designed the study. KC, K-IJ, and SL collected the data and performed the experiment, and KC analyzed the data and wrote the first draft of the manuscript. KC, S-HC, K-IJ, SL, and J-HC contributed to the final manuscript.

## Conflict of Interest Statement

The authors declare that the research was conducted in the absence of any commercial or financial relationships that could be construed as a potential conflict of interest.
