# Supplementary material for: Three Weeks of rTMS Treatment Maintains Clinical Improvement But Not Electrophysiological Changes in Patients With Depression: A 6-Week Follow-Up Pilot Study
Source: Front Psychiatry. 2019 Jun 7;10:351. doi: 10.3389/fpsyt.2019.00351 (PMC6566016; doi:10.3389/fpsyt.2019.00351)
Supplement: Supplementary file 1 [file DataSheet_1.docx]

Supplementary Material

Three weeks of rTMS treatment maintains clinical improvement but not electrophysiological changes in patients with depression: A 6-week follow-up study

***Kyung Mook Choi^1,2,3,4^, Soo-Hee Choi^5^, Sang Min Lee^2,3,4^, Kuk-In Jang^2,3,4^, and Jeong-Ho Chae^2,3,4*^***

*^1^Institute for Brain and Cognitive Engineering, Korea University, Seoul, South Korea*

*^2^Department of Psychiatry, Seoul St. Mary’s Hospital, Seoul, South Korea*

*^3^Institute of Biomedical Industry, The Catholic University of Korea, College of Medicine, Seoul, South Korea*

*^4^Department of Biomedicine & Health Sciences, The Catholic University of Korea, College of Medicine, Seoul, South Korea*

*^5^Department of Psychiatry, Seoul National University Hospital, Seoul, South Korea*

**Running Title**: rTMS maintenance effect in depression

***Correspondence:**Jeong-Ho Chae

alberto@catholic.ac.kr

# Correlation analysis

**1 Method**

The correlation analyses were performed for identifying the relationship between clinical rating scales and electrophysiological improvements. The correlation analysis (Pearson correlation coefficient) was performed for 3 weeks of measurement minus the baseline measurement and for 6 weeks of measurement minus the baseline measurement for the change in rating scales and P200 amplitudes (μV) and between the change in rating scales and P300 latencies (ms).

**2 Results**

In the correlation analyses (Fig 1 and 2, Table 1), in the case of P200 amplitude changes after 3 weeks of rTMS, FP1 correlated positively with the blaming others strategy of Cognitive Emotion Regulation Questionnaire (CERQ) (1), while Fz correlated negatively with the self-blame strategy and acceptance strategy of CERQ. FCz, correlated negatively with the self-blame strategy of CERQ, indicating that some scales of CERQ are related to the amplitude changes of the frontal channels. In brief, FP1 P200 amplitude increases with the improvement in the blaming others strategy, whereas Fz P200 amplitude decreases with the improvement in the self-blame and acceptance strategies. FCz P200 amplitude decreases with the improvement of the self-blame strategy. In the case of P300 latency changes after 3 weeks of rTMS, FP2 correlated negatively with the catastrophizing and blaming others strategies of CERQ, and Fz correlated negatively with the catastrophizing strategy of CERQ, indicating that FP2 P300 latency decreases with the improvement in the catastrophizing and blaming others strategies, and that Fz P300 latency decreases with the improvement in the catastrophizing strategy. Therefore, P200 amplitude and P300 latency in the frontal regions reflect CERQ’s blaming, acceptance, and catastrophizing strategy scores. In case of P300 latency changes after 6 weeks, Hamilton Anxiety Scale (HAM-A) correlated positively with Pz, Ruminative Response Scale (RRS) (2) correlated negatively with FP2, FCz, and Cz. The reappraisal of Emotion Regulation Questionnaire (ERQ) (3) correlated positively with FZ, FCz, and Cz, and the suppression correlated positively with FCz and Cz. The putting into perspective strategy of CERQ correlated positively with FP2, FCz, and Cz. The results indicate that after 6 weeks, anxiety symptoms increase as of Pz latency increases, rumination decreases with as FP2, FCz, and Cz latencies increase, the ERQ scores increase as FZ, FCz, and Cz latencies increase, and the putting into perspective strategy of CERQ increases as FP2, FCz, and Cz increase. The P300 latency changes in the 6 weeks after 3 weeks of rTMS reflect anxiety, rumination, and emotion regulation. The results showed that there was a correlation between the two measurements in the changes of some clinical ratings and channels.

**
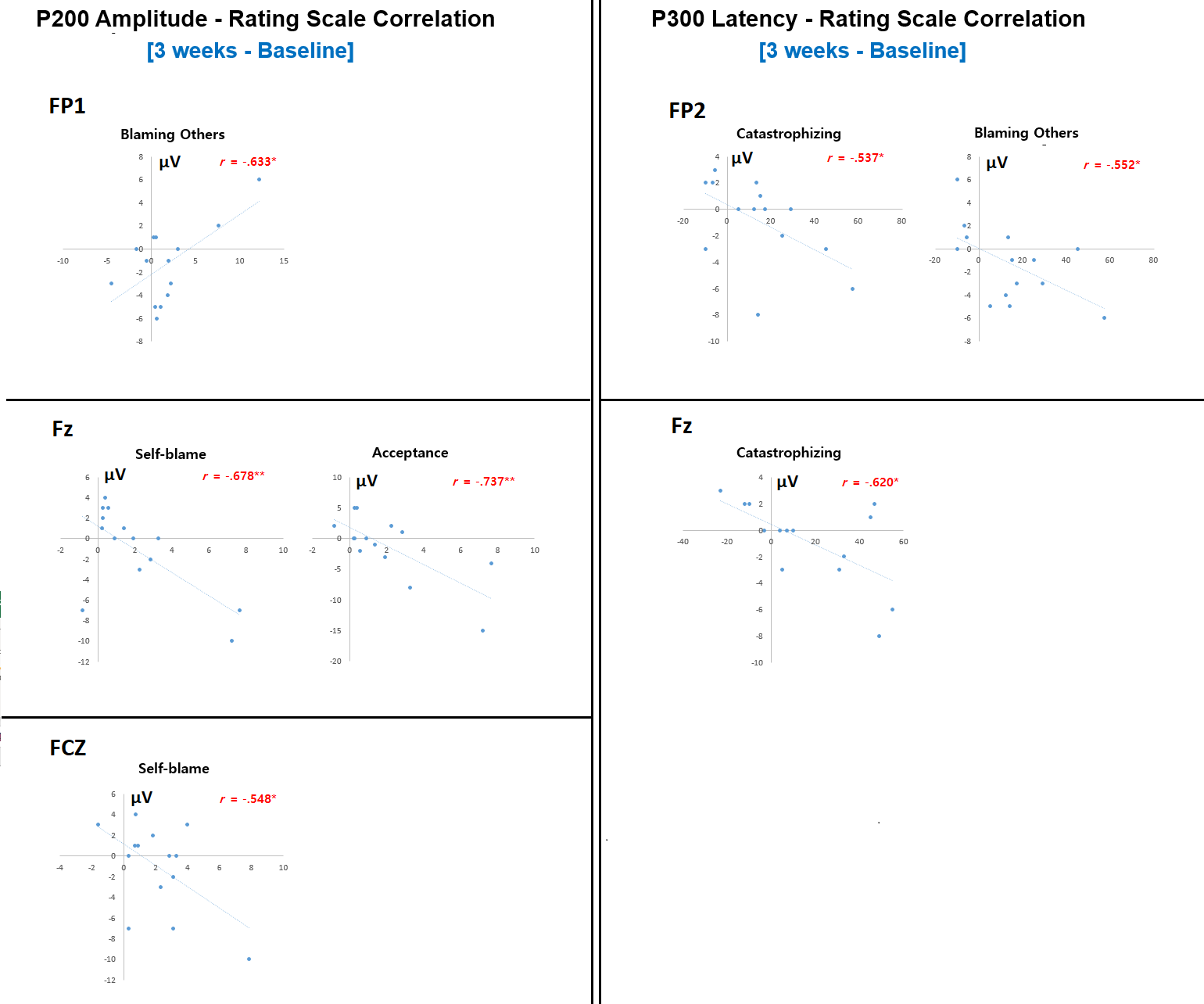
**

**Fig 1. Correlations of 3-weeks measurement minus baseline measurement between the change of rating scales and P200 amplitudes (μV) and between the change of rating scales and P300 latencies (ms).**

**. Correlation is significant at the 0.01 level (2-tailed).

*. Correlation is significant at the 0.05 level (2-tailed).

**
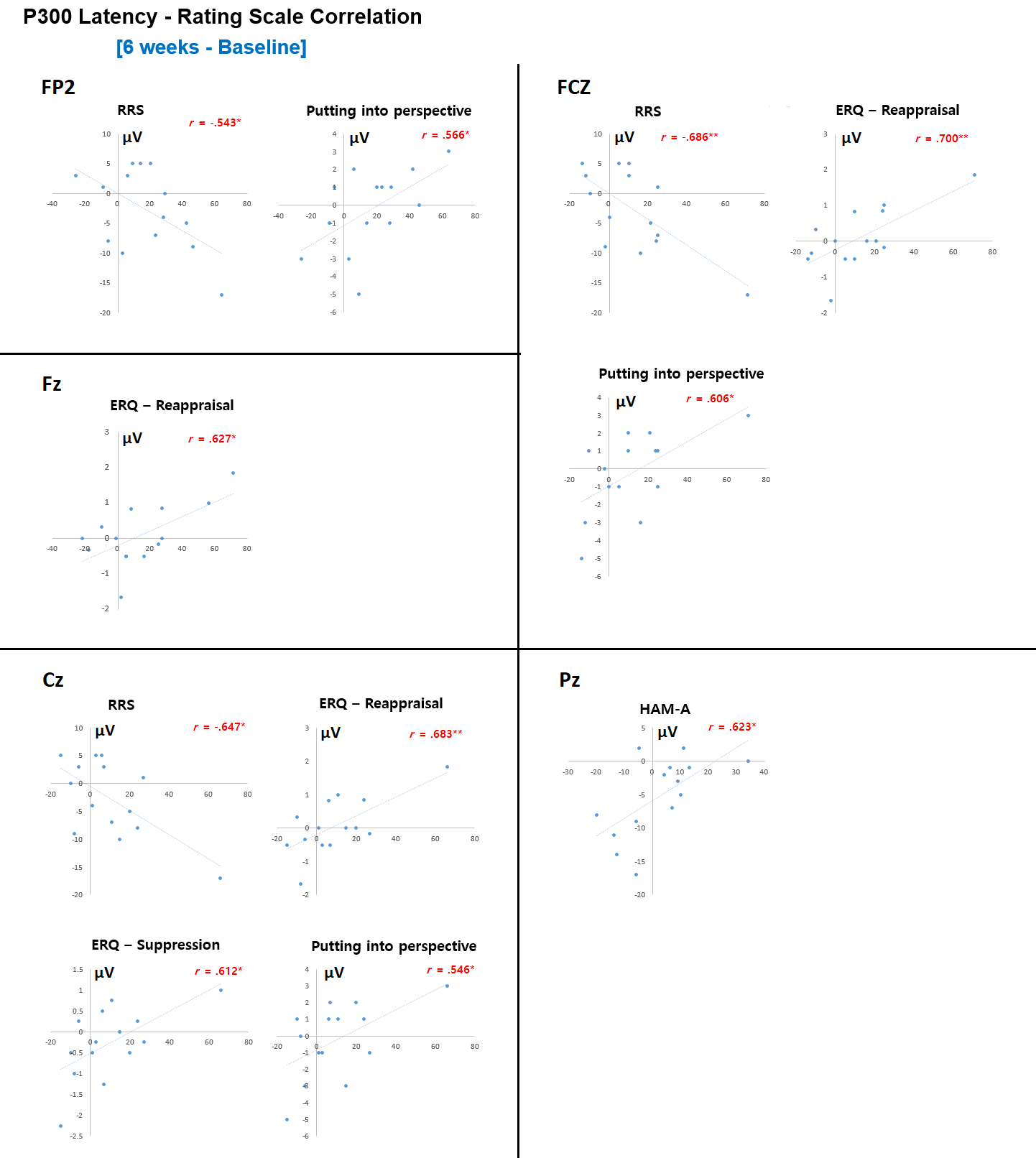
**

**Fig 2. Correlations of 6-weeks measurement minus the baseline measurement between the change of rating scales and P200 amplitudes (μV) and between the change of rating scales and P300 latencies (ms).**

**. Correlation is significant at the 0.01 level (2-tailed).

*. Correlation is significant at the 0.05 level (2-tailed).

**Table 1. Correlations of 3-weeks measurement minus the baseline measurement and of 6-weeks measurement between the change of rating scales and P200 amplitudes (μV) and between the change of rating scales and P300 latencies (ms)**

**3 weeks - baseline**

| **P200 Amplitude** |  |  |  |  |  |  |
| --- | --- | --- | --- | --- | --- | --- |
|  |  | **FP1** | **Fz** | **FCz** |  |  |
| **Self-blame** | *r* ^a^ | - | -.678** | -.548* |  |  |
|  | *p* | - | 0.008 | 0.042 |  |  |
|  | n | - | 14 | 14 |  |  |
| **Acceptance** | *r* ^a^ | - | -.737** | - |  |  |
|  | *p* | - | 0.003 | - |  |  |
|  | n | - | 14 | - |  |  |
| **Blaming others** | *r* ^a^ | .633* | - | - |  |  |
|  | *p* | 0.015 | - | - |  |  |
|  | n | 14 | - | - |  |  |

| **P300 Latency** |  |  |  |  |  |  |
| --- | --- | --- | --- | --- | --- | --- |
|  |  | **FP2** | **Fz** |  |  |  |
| **Catasrophizing** | *r* ^a^ | -.537* | -.620* |  |  |  |
|  | *p* | 0.048 | 0.018 |  |  |  |
|  | n | 14 | 14 |  |  |  |
| **Blaming others** | *r* ^a^ | -.552* | - |  |  |  |
|  | *p* | 0.041 | - |  |  |  |
|  | n | 14 | - |  |  |  |

**6 weeks - baseline**

| **P300 Latency** |  |  |  |  |  |  |
| --- | --- | --- | --- | --- | --- | --- |
|  |  | **FP2** | **Fz** | **FCz** | **Cz** | **Pz** |
| **HAM-A** | *r* ^a^ | - | - | - | - | .623* |
|  | *p* | - | - | - | - | 0.017 |
|  | n | - | - | - | - | 14 |
| **RRS** | *r* ^a^ | -.543* | - | -.686** | -.647* | - |
|  | *p* | 0.045 | - | 0.007 | 0.012 | - |
|  | n | 14 | - | 14 | 14 | - |
| **ERQ - Reappraisal** | *r* ^a^ | - | .627* | .700** | .683** | - |
|  | *p* | - | 0.016 | 0.005 | 0.007 | - |
|  | n | - | 14 | 14 | 14 | - |
| **ERQ - Suppression** | *r* ^a^ | - | - | .625* | .612* | - |
|  | *p* | - | - | 0.017 | 0.02 | - |
|  | n | - | - | 14 | 14 | - |
| **Putting into perspective** | *r* ^a^ | .566* | - | .606* | .546* | - |
|  | *p* | 0.035 | - | 0.022 | 0.043 | - |
|  | n | 14 | - | 14 | 14 | - |

**. Correlation is significant at the 0.01 level (2-tailed).

*. Correlation is significant at the 0.05 level (2-tailed).

**REFERENCES**

1. Garnefski N, Kraaij V, Spinhoven P. Negative life events, cognitive emotion regulation and emotional problems. Pers Individ Dif (2001) 30:1311–27. doi: 10.1016/S0191-8869(00)00113-6

2. Treynor W, Gonzalez R, Nolen-Hoeksema S. Rumination reconsidered: a psychometric analysis. Cogn Ther Res (2003) 27:247–59. DOI: 10.1023/A:1023910315561

3. Gross JJ, John OP. Individual differences in two emotion regulation processes: implications for affect, relationships, and well-being. J Pers Soc Psychol (2003) 85:348–62. doi: 10.1037/0022-3514.85.2.348
